# Sugar Cane (*Saccharum officinarum* L.) Waste Synthesized Si,N,S-Carbon Quantum Dots as High-Performance Corrosion Inhibitors for Mild Steel in Hydrochloric Acid

**DOI:** 10.1021/acsomega.4c05908

**Published:** 2024-12-12

**Authors:** Rayani da Silva Nunes, Victor Magno Paiva, Sanair Massafra de Oliveira, Clara Muniz da Silva de Almeida, Mariane Silva de Oliveira, Joyce Rodrigues de Araujo, Bráulio Soares Archanjo, Natasha Midori Suguihiro, Eliane D’Elia

**Affiliations:** † Department of Inorganic Chemistry, Universidade Federal do Rio de Janeiro UFRJ, Avenida Athos da Silveira Ramos, 149, Cidade Universitária, 21941-909 Rio de Janeiro, Brazil; ‡ Materials Metrology Division, Instituto Nacional de Metrologia, Qualidade e Tecnologia INMETRO, Avenida Nossa Sra. das Graças, 50, Xerém, 25250-020 Duque de Caxias, Brazil; § Department of Nanotecnology, Universidade Federal do Rio de Janeiro Campus UFRJ—Duque de Caxias Professor Geraldo Cidade, Rodovia Washington Luiz, 19593, 25240-005 Duque de Caxias, Brazil

## Abstract

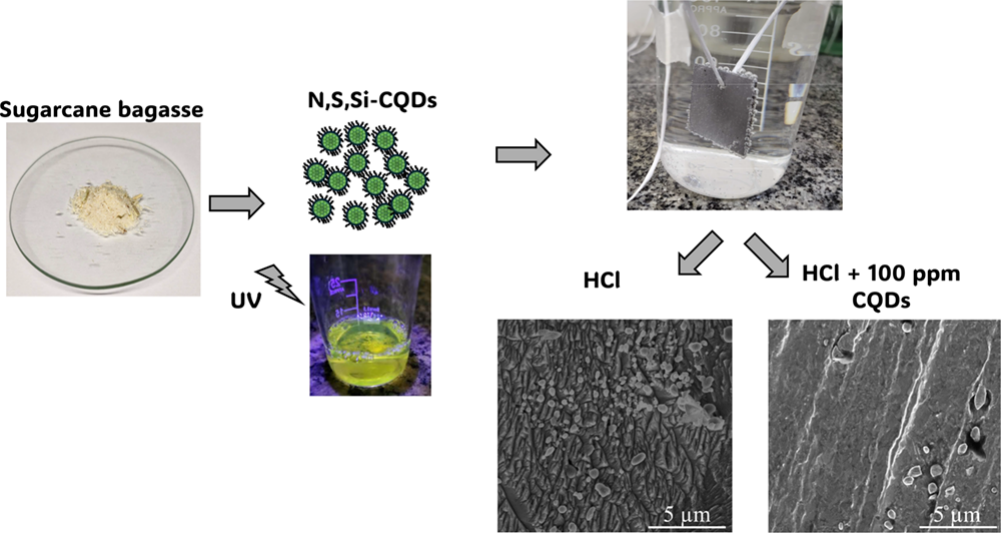

This work reports the obtention of Si,N,S-CQDs from sugar cane bagasse and their inhibitory action on the mild steel corrosion in 1 mol L^–1^ HCl solution. The CQDs were successfully obtained and characterized by X-ray photoelectron spectroscopy, Fourier-transform infrared spectroscopy, Dynamic light scattering, Raman, and UV–vis techniques, also showing endogenous self-doping. The anti-corrosive activity of CQDs was investigated by gravimetric tests, potentiodynamic polarization curves, electrochemical impedance measurements, atomic force microscopy, and scanning electron microscopy. The electrochemical results show that the CQDs present a predominant inhibitory action on the cathodic process, presenting inhibition efficiency of 82, 89, 91, and 94% for 15, 25, 50, and 100 ppm, respectively. Gravimetric tests varying temperature demonstrate that the inhibitor functions through physical adsorption and remains effective for up to 72 h, exhibiting corrosion efficiency of 80.2, 93.2, 96.3, and 97.8% at 15, 25, 50, and 100 ppm concentrations, respectively, after 72 h of immersion. Dynamic light scattering and zeta potential measurements indicate that agglomerations of CQDs play a crucial role in inhibiting corrosion. These results show an excellent alternative for using sugar cane bagasse to produce CQDs and its application as a corrosion inhibitor, valuing agricultural waste and simultaneously solving industry problems.

## 1 Introduction

Corrosion has stood out as one of the biggest industry problems. Corrosive processes have been responsible for high costs for the industry, whether caused by equipment maintenance, product recalls, or production stoppages when contamination, and for containing damages related to work or environmental accidents. Mild steel is recognized in industries for its good mechanical resistance, machinability, and low cost. However, it has low chemical resistance, especially in environments with high acidity. In the oil and gas industry, corrosion inhibitors are mainly used to mitigate corrosive processes related to the action of H_2_S, CO_2_, and acidic solutions that can be used in reservoir stimulation operations, removal of scale from pipes, or acid pickling. Hydrochloric acid is used most in these procedures due to its low cost.^1−9^

One of the most used methodologies to mitigate corrosion is corrosion inhibitors due to their cost-benefit, low cost, and easy application. Organic corrosion inhibitors act as protective films that adsorb on the metal surface, influencing the electrochemical processes that trigger corrosion. Adsorption inhibitors usually are organic compounds containing unsaturation and/or strongly polar groups containing nitrogen, oxygen, or sulfur.^10−13^

In this way, carbon quantum dots (CQDs) emerge as excellent candidates for corrosion inhibitors. CQDs are spherical nanoparticles smaller than 10 nm, with great potential in biomedicine, optoelectronics, chemical sensors, electrocatalysis, and corrosion protection. Among their various characteristics, such as low cost, low toxicity, stability, conductivity, good biocompatibility, and fluorescence, they are quite soluble in an aqueous medium, easily functionalized, and can be obtained from biomass, guaranteeing a high degree of self-doping (depending on the raw material).^13−19^ Due to these characteristics, CQDs have been applied as corrosion inhibitors for various media and substrates, presenting excellent corrosion efficiency values.^20−29^ However, studies on using CQDs from agro-industrial waste as corrosion inhibitors are still limited.

Raw materials from renewable sources are excellent candidates to act as precursors for new corrosion-inhibiting molecules. They are natural, cheap, abundant, and can be obtained from biomass conversion; they are rich in biomolecules with an abundance of C, N, O, S, and P, among other elements. In this context, using sugar cane bagasse as a raw material to obtain CQDs appears to be advantageous from an environmental point of view, as it becomes an alternative to using a residue/byproduct.

Sugar cane bagasse, obtained through sugar cane processing to produce ethanol and sugar cane juice, is the most considerable residue from the Brazilian agroindustry. For each ton of sugar cane ground in the industry, 700 L of sugar cane juice and 300 kg of bagasse (50% DM) are obtained. Therefore, 250 million tons of sugar cane are ground in Brazilian mills and distilleries, and each year, 75 million tons of sugar cane bagasse are obtained.^30^ Furthermore, sugar cane bagasse is rich in lignin, hemicellulose, and cellulose, which are precursors to produce carbon quantum dots and also present in its composition other bioactives that can induce endogenous self-doping to the CQDs produced.^31−33^

Due to its increasing potential use and the problems faced by the industry regarding corrosion and the environment, sugar cane bagasse was used as a raw material in producing carbon quantum dots, and its application as a corrosion inhibitor for mild steel in 1 mol L^–1^ HCl was evaluated.

## 2 Methods

### 2.1 Carbon Quantum Dots Synthesis

**Figure 1:**
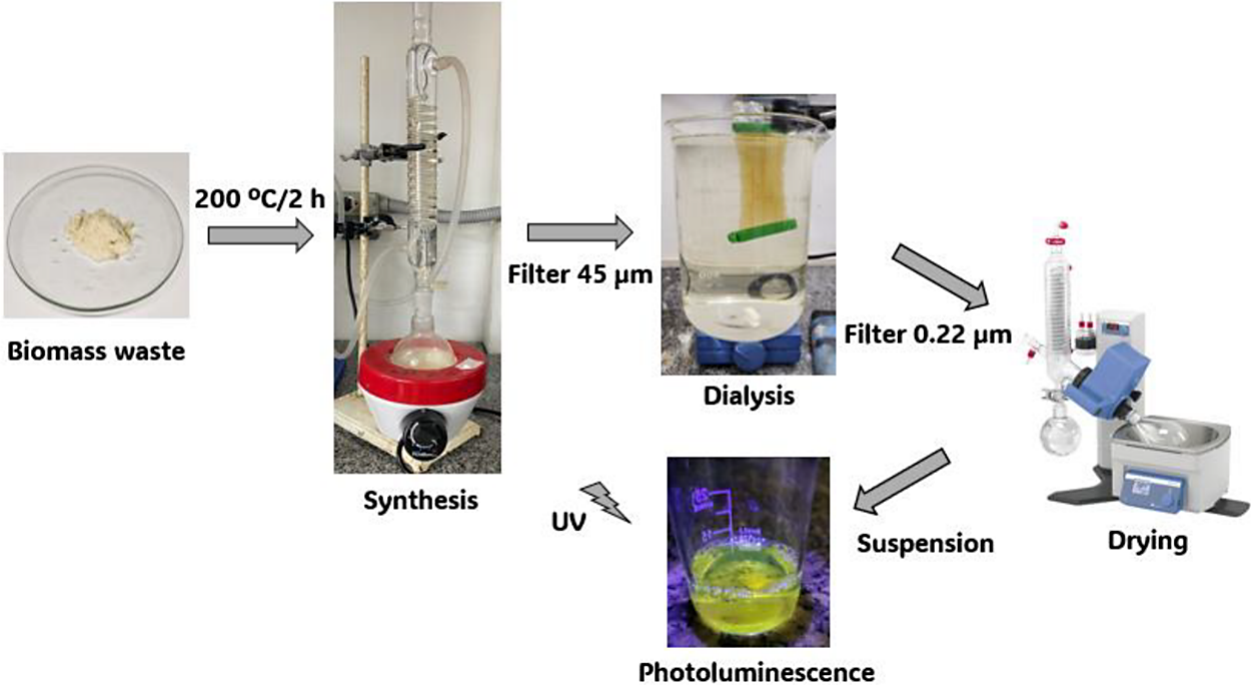
Simplified diagram of obtaining CQDs from sugar cane bagasse.

5 g of previously freeze-dried and crushed sugar cane bagasse was added to a synthesis flask containing 50 mL of 1 mol L^–1^ H_2_SO_4_ to synthesize carbon quantum dots. The mixture was placed in a heating blanket connected to reflux at 200 °C for 2 h. Then, the mixture was resuspended in 50 mL of water and filtered through a 45 μm membrane to remove solid sugar cane residue and a 0.22 μm membrane to discard large carbon particles. The synthesis procedure was performed twice, and then the synthesized products were mixed and dialyzed by a two kDa membrane size (Slide-A-Lyzer Dialysis Flasks, 250 mL) for 48 h to remove possible impurities. After dialysis, a rotary evaporator dried the solution, and a stock solution was prepared at a concentration of 2.5 mg mL^–1^, which was used throughout the study. In this methodology, the yield of CQDs obtained was approximately 25%, and 4 L of liquid waste were generated, which were neutralized and discarded. A simplified scheme of the CQDs synthesis is presented in Figure 1.

### 2.2 Characterization

The determination of the CQDs size was carried out using dynamic light scattering (DLS) technique. DLS analysis was performed using Anton Parr Litesizer 500 and CQDs samples at 0.2 mg mL^–1^ concentration in dimethylformamide. The chemical characterization of the particles obtained was studied using Raman, Fourier-transform infrared spectroscopy (FTIR), and X-ray photoelectron spectroscopy (XPS) techniques. Raman measurements were performed using Confocal Raman Microscope: SENTERRA II with 532 nm laser excitation. For the FTIR analysis, the Nicolet 6700FT-IR spectrometer was used in the range from 400 to 4000 cm^–1^. The XPS was carried out in an ultrahigh vacuum station (Escaplus P System, Omicron Nanotecnology, Taunusstein, Germany) with pressure in the measurement chamber of 10^–9^ mbar, using an Al X-ray source (Κα = 1486.7 eV) powered by 300 W. Survey spectra were obtained with 160 eV analyzer pass energy and 1.0 eV step size. C 1s, O 1s, N 1s, S 2p, and Si 2p high-resolution spectra were obtained with 30 eV pass energy in the analyzer and 0.05 eV steps. The peak fittings were performed with the CasaXPS software.

UV–vis and Fluorescence Spectroscopy techniques were used to characterize the photochemical properties. The UV–vis spectrum was determined in the 200–700 nm range using a UV-1800 spectrophotometer (Shimadzu), while the Fluorescence Spectrum was studied using the RF 5301 cuvette fluorimeter (Shimadzu). Fluorometry analysis was performed using the following excitation wavelengths: 300, 310, 320, 330, 340, 350, 360, 370, 380, 390, 400, and 500 nm. A water suspension was used for both analyses at 0.2 mg mL^–1^ for UV–vis and 1 mg mL^–1^ for Fluorescence Spectroscopy.

Surface characterization of the mild steel before and after the corrosion test was conducted by scanning electron microscopy (SEM) using a Scanning Electron Microscope Magellan 450 and atomic force microscopy (AFM) using JPK NanoWizard NanoScience techniques.

### 2.3 Surface Preparation of Mild Steel

The study used mild steel, whose composition, in weight %, is C: 0.18, P: 0.04, S: 0.05, Mn: 0.30, Si: trace, and Fe: remainder. Both gravimetric and electrochemical tests involved specimens subjected to the same pretreatment. This pretreatment consisted of abrading the surface using 100, 320, and 600 mesh abrasives on an AROPOL 2 V polisher (AROTEC), followed by washing with deionized water and acetone and subsequent drying. The surface area for the gravimetric test was approximately 13 cm^2^, while the electrochemical test was 0.70 cm^2^.

### 2.4 Gravimetric Measurements

In gravimetric tests, the specimens were immersed in an acid solution (HCl 1 mol L^–1^) for 2, 24, 48, and 72 h in the presence and absence of the inhibitor. The effect of inhibitor concentration on the corrosion rate of mild steel was evaluated at 15, 25, 50, and 100 ppm. To determine the corrosion rate (*W*_corr_ in mg g^–2^ h^–1^), the mass variation before and after the tests was used in eq 1, and then the inhibition efficiency (IE%) was used through eq 2.
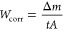
1where Δ*m*, *t*, and *A* correspond to the change in mass, the immersion time, and the specimen area, respectively. To determine the inhibition efficiency (IE%), the following equation is used:
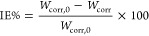
2where *W*_corr,0_ and *W*_corr_ are the corrosion rate in the absence and presence of inhibitor, respectively.

The effect of temperature on the corrosion rate was also evaluated in the absence and presence of the inhibitor at 25 ppm for a 2-h immersion time at 35, 45, 55, and 65 °C to study the effect of temperature and to determine the thermodynamic parameters of activation on the inhibitory process.

### 2.5 Electrochemical Measurements

Electrochemical measurements were conducted using a 3-electrode electrochemical cell. This included mild steel as a working electrode, with a surface area of approximately 0.70 cm^2^, a saturated calomel electrode (SCE) as reference, and a platinum counter electrode. The tests were carried out at a temperature of 25 °C, under natural aeration conditions, both in the absence and presence of an inhibitor, at concentrations of 15, 25, 50, and 100 ppm.

The electrochemical measurements, open circuit potential, potentiodynamic polarization, and electrochemical impedance spectroscopy (EIS) were carried out using an AUTOLAB PGSTAT 128 N potentiostat (Netherlands). After 2 h, the mild steel electrode reached its stable open circuit potential (OCP). Electrochemical impedance measurements were acquired using a 10 mV amplitude sine wave at a frequency range from 10^5^ to 10^–2^ Hz. Polarization curves were acquired with a scan rate of 1 mV s^–1^ from −300 to 300 V relative to the stable OCP.

Eqs 3 and 4 were used to calculate the inhibition efficiency using the EIS and potentiodynamic polarization curve data.
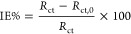
3where *R*_ct,0_ is the charge transfer resistance in the absence of inhibitor and *R*_ct_ is the charge transfer resistance in the presence of the inhibitor.
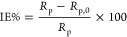
4where *R*_p,0_ and *R*_p_ are the polarization resistance of the mild steel in the absence and presence of the inhibitor, respectively.

## 3 Results and Discussion

### 3.1 Carbon Quantum Dots Characterization

**Figure 2:**
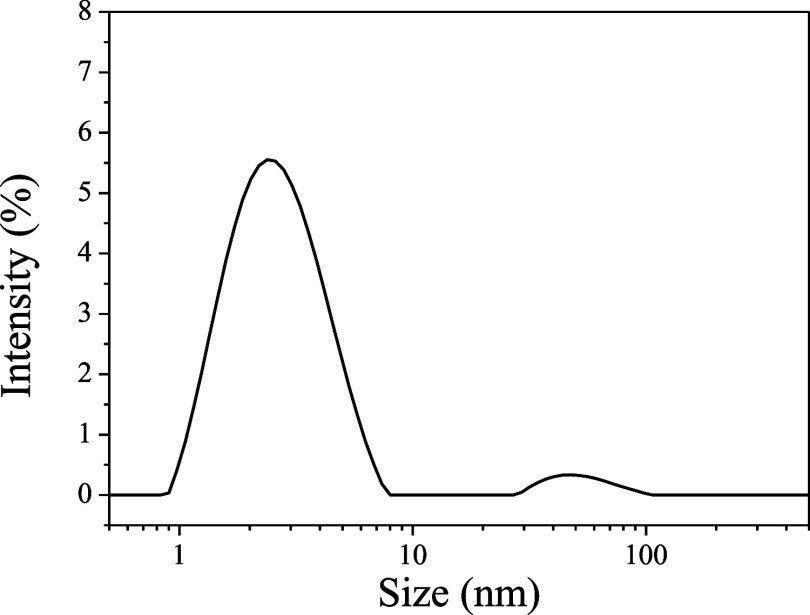
Dynamic light scattering (DLS) size distribution of CQDs obtained from sugar cane bagasse in the concentration of 0.02 mg mL^–1^ in DMF.

One of the most important physical properties of CQDs is their size. Particles smaller than 10 nm are typically classified as carbon quantum dots.^34,35^ As shown in Figure 2, the obtained CQDs have a hydrodynamic size of 2.79 nm, a narrow size distribution, and a polydispersity index of 0.27 in DMF. Additionally, a small amount of agglomerates cover an area of 3.6%.

**Figure 3:**
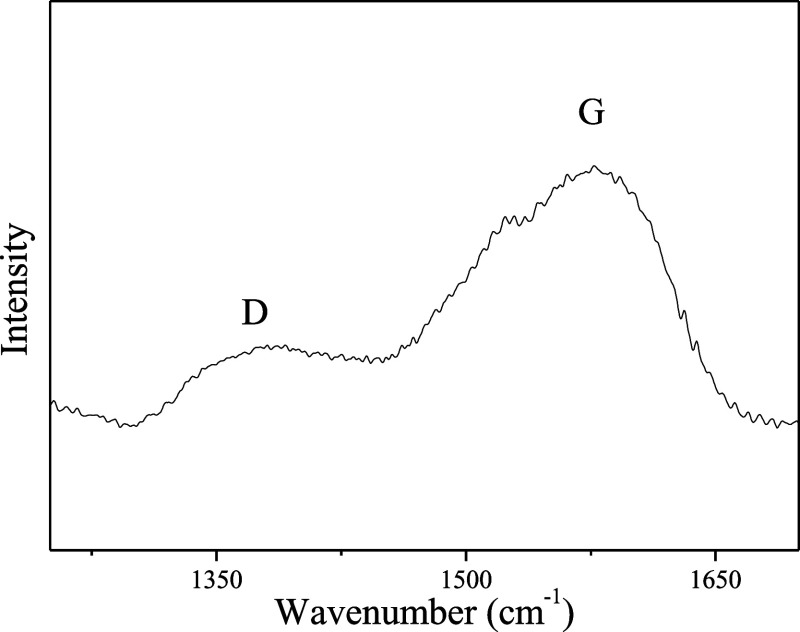
Raman spectrum of the CQDs obtained from the sugar cane bagasse.

The Raman spectrum of the CQDs obtained (Figure 3) presents a typical characteristic of carbon structure, presenting the D band (disorder) related to disorder and defects in the structure and the G band (graphitic), associated with the stretching of the C–C bond in graphitic materials, and is expected to all sp^2^ carbon systems.^36−38^ The broad peaks obtained corroborate the DLS result since broad peaks are typical characteristics of nanosized carbon particles.^39^ Also, the spectrum indicates that the particles obtained are composed of a mix of carbon sp^2^ and sp^3^, which consist of functionalized carbon quantum dot structures. The carbon structure will be evaluated in depth using FTIR and XPS techniques.

**Figure 4:**
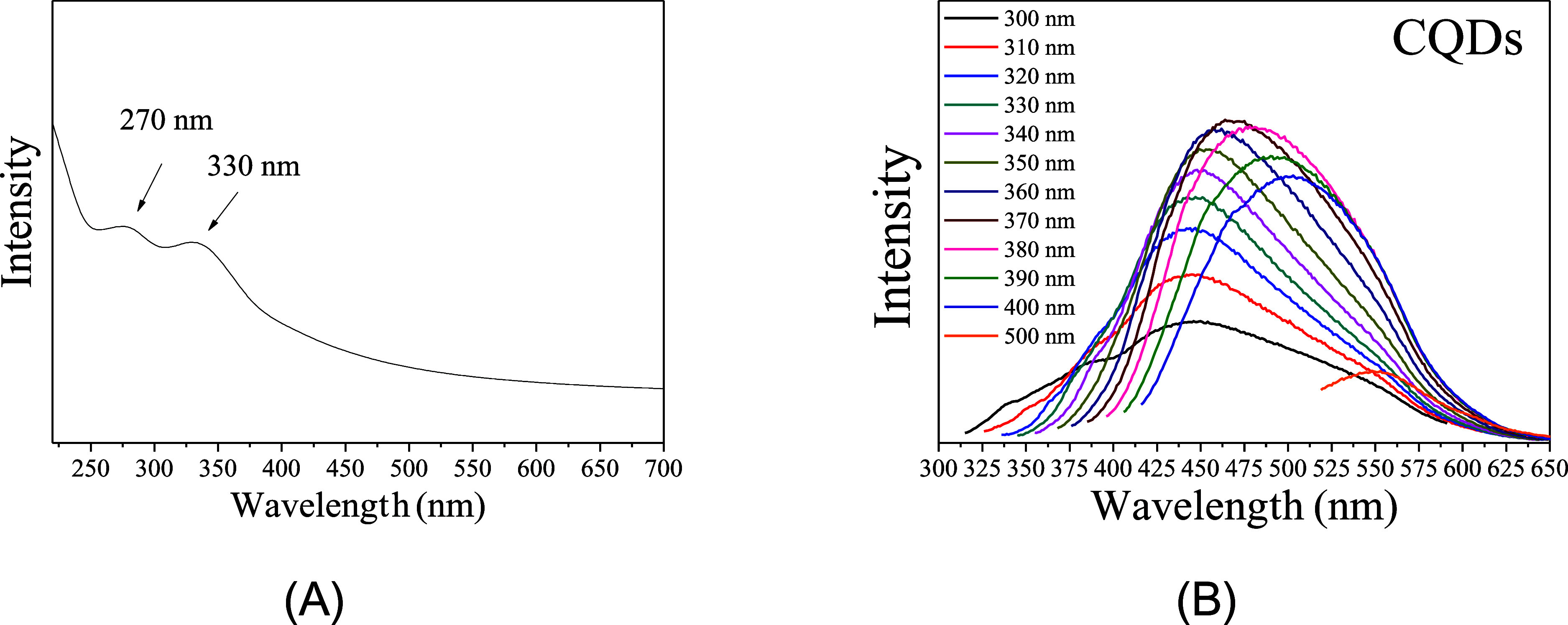
(A) UV–vis absorption spectrum and (B) the emission spectrum profile in different emission wavelengths of the CQDs at 0.2 and 1.0 mg mL^–1^, respectively.

One of the characteristics that most attracted research into carbon quantum dots is their luminescent properties. The UV–vis absorption spectrum of the particles obtained is shown in Figure 4A. The CQDs presented two well-defined bands at wavelengths around 330 and 270 nm and elongation from 600 nm. The absorption at 270 nm can be attributed to the π–π* transition of aromatic C = C bonds of the graphitic domains present in the particles, and at 330 nm, this absorption band is related to the n-π* transition of C=O/C–N/C=N groups. The elongation of the spectrum may be associated with the functional groups attached to the CQDs surface.^37,40−43^Figure 4B shows the fluorescence spectrum of CQDs at different excitation wavelengths. As can be seen for all CQDs obtained, the photoluminescence peak is shifted toward the red region as the excitation wavelength increases, showing a strong dependence between the emission and excitation wavelength, a characteristic similar to other CQDs present in the literature.^17,44,45^ By interpreting the results, it was observed that the fluorescence intensity was linearly dependent on the excitation wavelength, and the highest emission was obtained at 464 nm with λ_exc_ = 370 nm. These results corroborate that the size of CQDs measured by DLS analysis (Figure 2) is accurate, as only CQDs smaller than about 5 nm^46^ are expected to fall within the blue spectrum.

**Figure 5:**
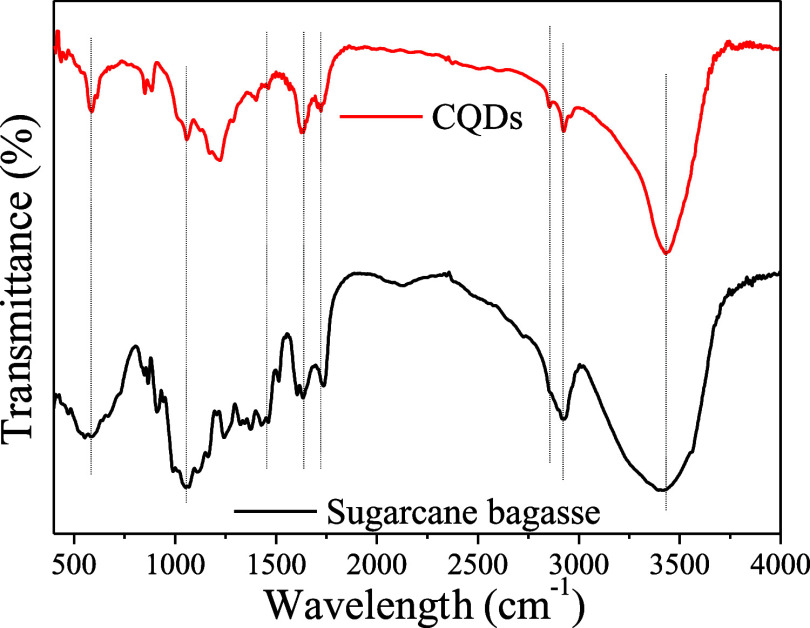
FTIR spectra of the sugar cane bagasse and sugar cane bagasse derived CQDs.

Once CQDs were obtained, the FTIR and XPS techniques further investigated the chemical composition and functional groups in CQDs. As observed by the FTIR spectra (Figure 5), the CQDs and sugar cane bagasse present very similar spectra, giving evidence of an endogenous self-doping, showing that the CQDs produced incorporated chemical structures of the precursor, presenting several functional groups on their surface. As shown in Figure 5, the FTIR spectra of CQDs present well-defined bands, among them the broad peak at 3400 related to the N–H/O–H stretching vibration. The two bands at 2920 and 2855 cm^–1^ correspond to the vibrations of saturated C–H. The stretching vibration of C=O, C=C, C–H/C-N, C–O, and C=S can be found at the absorption bands 1720, 1634, 1461, 1400, and 1297 cm^–1^, respectively. The absorption bands at 1056 and 851 cm^–1^ were ascribed to the C–S and Si–O–Si stretching vibrations.^47−52^

**Figure 6:**
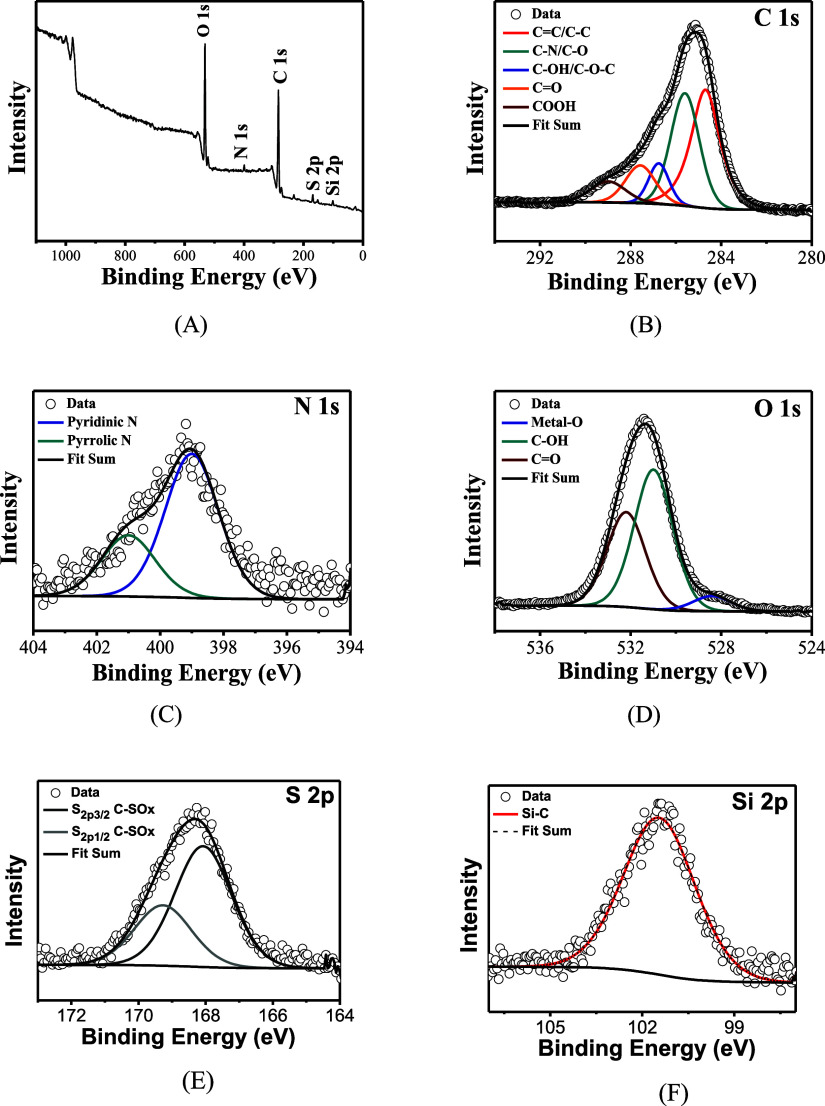
XPS spectra of carbon quantum dots obtained from sugar cane bagasse, where (A) survey spectrum and high-resolution spectra for (B) C 1s, (C) N 1s, (D) O 1s, (E) S 2p, and (F) Si 2p.

The XPS measurement showed the presence of C, O, N, S, Si, and Ca in the produced CQDs, as seen in the survey spectrum (Figure 6A). High-resolution deconvoluted spectra for C 1s, N 1s, O 1s, S 2p, and Si 2p are presented in Figure 6B–F, respectively. The C 1s spectrum (Figure 6B) shows five peaks at 284.3, 285.2, 286.3, 287.4, and 288.9 eV that can be attributed to the bonding energy of C=C/C–C, C–N/C–O, C–OH/C–O–C, C=O, and COOH, respectively. The presence of pyridinic and pyrrolic N is confirmed through N 1s spectrum (Figure 6C), which showed peaks at 398.9 and 400.7 eV, respectively. O 1s spectrum (Figure 6D) shows 528.5, 530.9, and 532.2 eV peaks, indicating the presence of metal-oxygen bonding, C–OH, and C=O, respectively. Finally, the S 2p spectrum (Figure 6E) presents two peaks at 168.1 and 169.3 eV assigned to S_2p3/2_ C–SO_*x*_ and S_2p1/2_ C–SO_*x*_ and Si 2p spectrum (Figure 6F) indicates the presence of Si–C (101.5 eV).^47−49,53−55^

These results show that the material obtained is mainly composed of sp^2^ and sp^3^ carbon, corroborating the RAMAN result, which presented the presence of D and G bands. They also confirm that the material obtained is rich in functional groups, mainly in N and O, which was already observed in UV–vis and FTIR analyses, where it was possible to observe a shift in the absorption spectrum due to the presence of functional groups and the presence of several bands in the FTIR spectrum.

The analysis also confirms endogenous self-doping. Sugar cane bagasse is mainly composed of carbohydrates (primarily hemicellulose, cellulose, and lignin) and fibers; depending on the type of analysis, it can reach 90% carbohydrates and around 60 and 38% for neutral and acidic fibers, respectively. Finally, the protein content is only around 2%.^56−58^ These values corroborate those obtained by XPS, which shows a material rich in carbon and oxygen, the elemental composition of carbohydrates. At the same time, nitrogen can come from fibers and proteins and sulfur from proteins. It is essential to highlight that sulfur may also have been introduced into the particle structure by the presence of sulfuric acid. Finally, Si is present in the inorganic composition of sugar cane bagasse.^59,60^

These results and other characterization techniques show that endogenous self-doping carbon quantum dots were successfully obtained from the acid hydrolysis of sugar cane bagasse. They show a high degree of functionalization, indicating that they are promising for application as a corrosion inhibitor.

### 3.2 Gravimetric Measurements

Table 1 presents the results of corrosion efficiency (IE) and corrosion rate (*W*_corr_) obtained from mass loss tests for mild steel in 1 mol L^–1^ HCl using CQDs at concentrations of 15, 25, 50, and 100 ppm and immersion times of 2, 24, 48, and 72 h.

**Table 1:** Gravimetric Measurements for the Mild Steel in 1 mol L^–1^ HCl in the Presence and Absence of CQDs at 2, 24, 48, and 72 h at a Temperature of 25 °C

		2 h		24 h		48 h	72 h		
Amostra	[CQDs] (ppm)	*W*_corr_ (mg cm^–2^ h^–1^)	IE (%)	*W*_corr_ (mg cm^–2^ h^–1^)	IE (%)	*W*_corr_ (mg cm^–2^ h^–1^)	IE (%)	*W*_corr_ (mg cm^–2^ h^–1^)	IE (%)
blank	0	1.87		1.88		1.39		0.934	
CQDs	15	1.09	41.7 ± 2.27	0.151	92.0 ± 1.91	0.122	93.5 ± 2.91	0.370	80.2 ± 6.91
	25	0.667	64.3 ± 0.94	0.140	92.6 ± 0.28	0.0740	96.1 ± 1.42	0.127	93.2 ± 3.69
	50	0.452	75.8 ± 0.52	0.133	92.9 ± 0.97	0.0623	96.7 ± 0.07	0.0692	96.3 ± 0.19
	100	0.360	80.7 ± 0.54	0.112	94.0 ± 0.81	0.0730	96.1 ± 0.01	0.0422	97.8 ± 0.80

As shown in Table 1, the corrosion rate (*W*_corr_) in the presence of CQDs demonstrated a significant decrease as both the concentration and immersion time increased compared to the blank, suggesting a notable inhibitory effect on the corrosion of mild steel in a solution of 1 mol L^–1^ of HCl. It is observed that after 24 h, the inhibitor was saturated with a concentration of 15 ppm.

Furthermore, it is observed that CQDs maintain their inhibition efficiency above 90% for concentrations of 15 ppm for 24 and 48 h. It showed inhibition efficiency above 90% for concentrations above 25 ppm for 72 h. These results demonstrate the continuous effectiveness of the inhibitor over the time studied, maintaining corrosion efficiency above 90%, showing the great potential for using the particles as a corrosion inhibitor under the conditions studied. Table 2 compares the anticorrosive efficiency of the CQDs produced in this work with others in the literature.

**Table 2:** Comparison of the Maximum Anticorrosive Efficiency of Sugarcane Bagasse CQDs with Other Carbon Quantum Dots Present in the Literature for Mild Steel in 1 mol L^–1^ HCl

material	optimum concentration (ppm)	maximum efficiency (%)	ref
*Zanthoxylum bungeanum* leaves derived CQDs	200	95.6	(22)
amino-guanidine and citric acid derived N-CQDs	200	95.3	(20)
grapefruit peels derived CQDs	200	94.1	(61)
citric acid and thiourea derived N,S-CQDs	400	96.6	(24)
methacrylic acid and *n*-butylamine derived N-CQDs	200	94.9	(62)
pumpkin seeds derived N,P,S-CQDs	10	94.6	(13)
pitaya peels derived N,S-CQDs	200	95.6	(63)
lupine derived N-CQDs	175	89.3	(64)
sugar cane bagasse derived N,S,Si-CQDs	25	96.1	this work

As Table 2 presents, many research groups are developing corrosion inhibitors from CQDs, which have produced good anti-corrosive efficiency results. Although all CQDs, their chemical composition directly influences their anticorrosive properties. As can be seen, the CQDs produced in this work obtained an extremely satisfactory anti-corrosion performance. Presenting a corrosion efficiency approximately equal to the highlighted works but with a much lower concentration, except the CQDs obtained from pumpkin seed.

To understand the interaction between mild steel and CQDs and their stability at different temperatures, immersion tests were carried out using 25 ppm of CQDs at 25, 35, 45, and 55 °C for 2 h (Table 3).

**Table 3:** Gravimetric Measurements for Mild Steel in 1 mol L^–1^ HCl in the Absence and Presence of 25 ppm of CQDs Over Two Hours Using Different Temperatures (25, 35, 45, and 55 °C)

	25 °C		35 °C		45 °C		55 °C	
sample	*W*_corr_ (mg cm^–2^ h^–1^)	IE (%)	*W*_corr_ (mg cm^–2^ h^–1^)	IE (%)	*W*_corr_ (mg cm^–2^ h^–1^)	IE (%)	*W*_corr_ (mg cm^–2^ h^–1^)	IE (%)
blank	1.87		3.13		5.48		10.3	
CQDs	0.667	64.3 ± 0.94	0.899	71.6 ± 0.76	3.00	45.3 ± 1.01	6.00	41.7 ± 0.62

Table 3 shows that increasing temperature significantly increases the corrosion rate (*W*_corr_) for inhibited and uninhibited systems. At the same time, the inhibition efficiency (IE) decreases when the temperature varies from 35 to 55 °C.

According to Popova et al.,^65^ there are three types of corrosion inhibitors. Those in which IE increases with temperature and the apparent activation energy (*E*_a_) decreases with the addition of the inhibitor. A chemical interaction between the inhibitory molecules and the metal surface is considered in this case. The second are those in which IE decreases with temperature, and *E*_a_ increases with the addition of the inhibitor. A physical interaction between the inhibitory molecules and the metal is considered in this case. The third is a rare type, in which IE remains constant with temperature (in this case, it is a pure blocking type inhibitor, acting only through superficial shielding).

**Figure 7:**
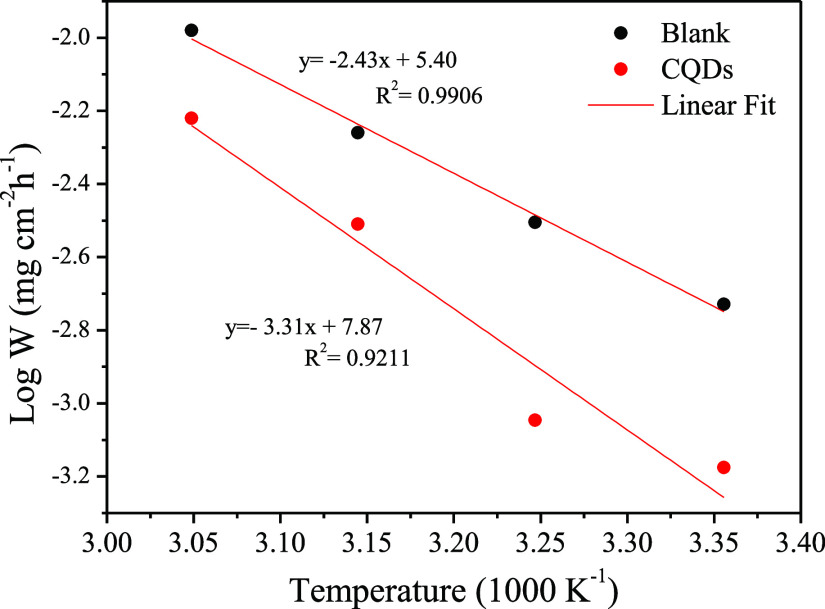
Arrhenius plot for mild steel in 1 mol L^–1^ HCl in the absence and presence of 25 ppm of CQDs at temperatures of 25, 35, 45, and 55 °C during 2 h of immersion.

Figure 7 shows the Arrhenius plots for mild steel corrosion in uninhibited and inhibited systems. The Arrhenius equation 5 could determine the activation energy
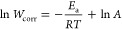
5where *W*_corr_ is the corrosion rate, *R* is the gas constant, *E*_a_ is the apparent activation energy and *T* is the temperature in Kelvin.

Using the angular coefficient of the equation, *E*_a_ values of 46.52 and 63.38 kJ mol^–1^ were obtained in the absence and presence of the inhibitor, respectively. The higher activation energy value found in the presence of CQDs indicates the physisorption of the inhibitor, which occurs through long-range forces (van der Waals forces) between adsorbate–adsorbent. They are nonspecific adsorption, easily reversible, and decrease adsorption with increasing temperature.^66,67^

Physisorption can be attributed to the Coulomb interaction between protonated inhibitor particles due to the acidic environment. As for physical adsorption, it can be postulated that the anions present (in this case, chloride ions) were initially adsorbed on the metal surface, giving it a negative charge, followed by the electrostatic adsorption of protonated inhibitor particles on the mild steel surface.^68^

### 3.3 Electrochemical Measurements

Electrochemical studies are essential tools that can provide information beyond inhibition efficiency, such as inhibitor type (cathodic, anodic, or mixed) and mechanism studies.

**Figure 8:**
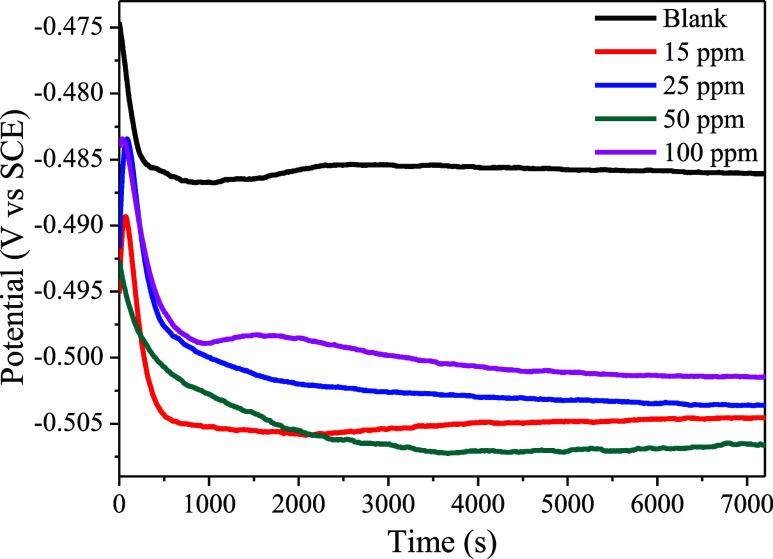
Open circuit potential behavior for mild steel in 1 mol L^–1^ HCl in the absence and presence of different concentrations of CQDs.

Figure 8 shows the variation in open circuit potential (OCP) for mild steel in 1 mol L^–1^ HCl over 2 h in the absence and presence of the inhibitor. An abrupt change in potential can be noticed in the first minutes of immersion, with an increase in value that later stabilizes at more negative values in the presence of inhibitors. The stabilized OCP for the uninhibited system reaches a value of −0.486 V vs. SCE, while for concentrations of 15, 25, 50, and 100 ppm, the OCP reaches −0.505, −0.504, −0.506, and −0.501 V vs SCE, respectively. The shift in OCP value due to the presence of the different concentrations of de CQDs is at most −20 mV, implying that the inhibitor is probably of the mixed type, which will be evaluated in the electrochemical polarization study. Observing the results, it is possible to notice that the open circuit potential reaches stability at approximately 3000 s.

The successive addition of CQDs to the medium decreases the OCP, probably due to the action of inhibitory particles, which indicates the formation of a protective film on the surface of the mild steel. OCP deviations to more negative values indicate that the CQDs act predominantly on the cathodic reaction.

**Figure 9:**
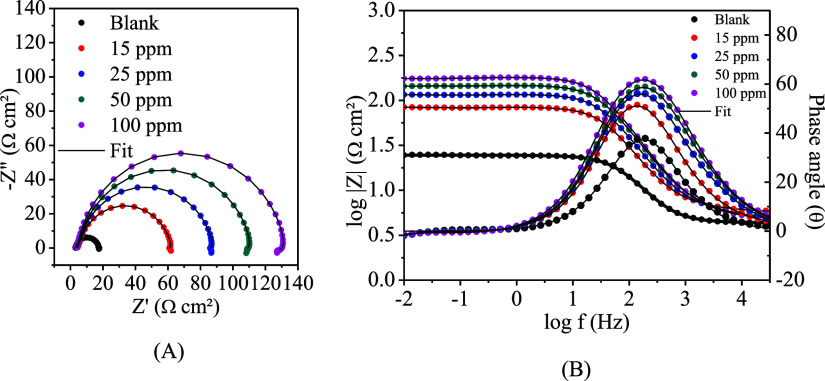
Nyquist diagrams (A) and Bode plots (B) of the mild steel in HCl 1 mol L^–1^ in the absence and presence of different concentrations of CQDs.

After stabilizing the OCP, EIS measurements and potentiodynamic polarization curves were performed. Figure 9 presents the Nyquist diagrams (Figure 9A) and Bode plots (Figure 9B) obtained for mild steel in a 1 mol L^–1^ HCl solution in the absence and presence of carbon quantum dots.

In the same way that it was observed in the gravimetric tests, the high concentration of the inhibitor results in corrosion attenuation since the increase in the number of particles with inhibitory capacity available for adsorption on the metal surface reduces the free area of metal/solution, decreasing thus the occurrence of the corrosive process.

According to the Nyquist diagrams (Figure 9A), it is possible to note that in the absence of CQDs, the diagram only presents a capacitive loop associated with the charge transfer resistance and the relaxation of the electrical double layer. With the addition of the inhibitor to the medium, there is an increase in the capacitive loop, indicating an increase in charge transfer resistance that is related to the inhibitor since the capacitive loop increases with the successive increase in the concentration of the CQDs, giving evidence of the formation of a protective film.^69,70^ The increase in the concentration of CQDs causes an inductive behavior to appear in the lower frequency range. This behavior is more apparent at 100 ppm. This inductive loop’s origin is probably the inhibitory particles’s relaxation process on the mild steel surface.

Adding carbon quantum dots in HCl solution 1 mol L^–1^ increased impedance modulus and phase angles in the Bode plots (Figure 9B) compared to the blank test. In the bode plot, a one-time constant is observed in the absence of the inhibitor in the 10,000–10 Hz region associated with the charge transfer process that shifts to lower frequencies with the addition of the CQDs. Higher phase angles were obtained in the presence of CQDs, suggesting that a protective film is formed in these conditions.

**Figure 10:**
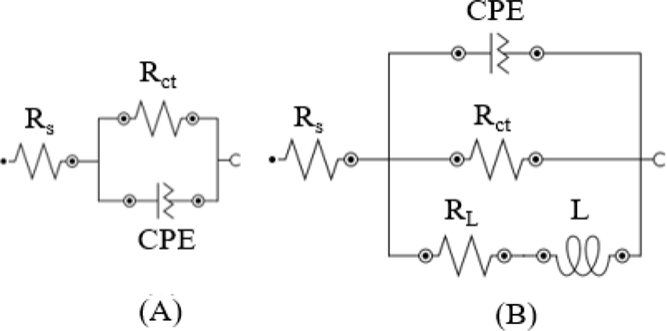
Equivalent circuit used to fit the experimental data obtained from the EIS (Figure 9) of the MS in 1 mol L^–1^ HCl (A) and in 1 mol L^–1^ HCl in the presence of the CQDs (B).

The electrochemical parameters were obtained from the electrochemical impedance diagrams using the electrochemical circuits presented in Figure 10, where CPE corresponds to the constant phase element, *R*_ct_ represents the charge transfer resistance, *L* and *R*_L_ represent the inductive elements, and *R*_s_ correspond to the solution resistance. The results are shown in Table 4. According to eq 3, the inhibition efficiency was calculated using the *R*_ct_ in the presence and absence of CQDs.

**Table 4:** Electrochemical Data Obtained from the EIS Equivalent Circuit for Mild Steel in a 1 mol L^–1^ HCl Solution, With and Without CQDs

inhibitor concentration (ppm)	*C*_dl_ (μF cm^–2^)	*L* (H cm^2^)	*R*_L_ (Ω cm^2^)	*R*_s_ (Ω cm^2^)	*R*_p_ (Ω cm^2^)	*R*_ct_ (Ω cm^2^)	IE (%)	χ^2^ (10^–3^)
0 (Blank)	160			5.56	14.4	14.4		1.7
15	81.9	0.63	1.58	5.59	59.55	60.5	76.2 ± 1.74	1.3
25	59.6	1.24	2.41	5.18	82.63	83.59	82.8 ± 0.65	0.072
50	49.9	1.58	2.79	4.95	100.85	102.9	86.0 ± 0.86	1.19
100	46.8	2.19	3.51	5.13	127.5	132.01	88.8 ± 0.37	0.081

The electrical double layer (*C*_dl_) was obtained from the equivalent circuit using the equation^71^ (6) below:
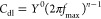
6where *f*_max_ is the frequency in which the imaginary component of the impedance is maximum, *Y*^0^ is the magnitude of the CPE, and *n* is the CPE exponent.^72^

The increase in CQD concentration reduces the capacitance of the electrical double layer (*C*_dl_) and increases the charge transfer resistance. The capacitance of the electrical double layer indicates the accumulation of charge between the electrolyte and the metal, and the drop in the local dielectric constant can explain its reduction.^73−75^

The increase in charge transfer resistance (*R*_ct_) can be explained by forming a protective film at the interface between the metal and the solution. These results indicate a decrease in the active area on the metal surface due to the adsorption of inhibitor molecules as the concentration of inhibitors increases.

**Figure 11:**
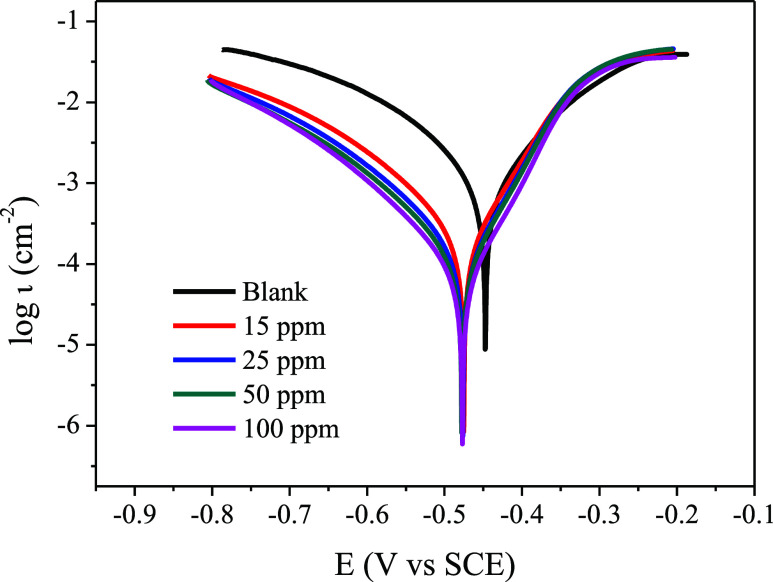
Potentiodynamic polarization curves for mild steel in 1 mol L^–1^ HCl in the absence and presence of CQDs.

Polarization is essential to understanding whether the inhibitor mitigates anodic, cathodic, or both reactions. Figure 11 shows the potentiodynamic polarization curves for mild steel in 1 mol L^–1^ HCl in the absence and presence of carbon quantum dots at room temperature.

A significant drop in cathodic current density is noted with adding CQDs to the medium, showing that the studied inhibitor acts predominantly on cathodic reactions. This corroborates what was observed in the OCP profile of the mild steel behavior (Figure 8) in the presence of the inhibitor. Since a reliable Tafel region is not seen in most of the polarization curves, the Tafel extrapolation method was not used. From the polarization curves, it was possible to obtain the polarization resistance values obtained from the linear polarization method using ±10 mV vs E_corr_ and inhibition efficiency (IE%). These parameters are provided in Table 5.

**Table 5:** Polarization Resistance Data from Polarization Curves for Mild Steel in Hydrochloric Acid in the Presence and Absence of CQDs

inhibitor (ppm)	OCP (mV/ECS)	*E*_corr_ (mV/ECS)	*R*_p_ (Ω cm^2^)	IE (%)
Branco	–486	–446	24.8	
15	–505	–477	92.5	73.2 ± 6.85
25	–504	–477	134	81.5 ± 0.80
50	–506	–481	176	85.9 ± 0.04
100	–501	–476	241	89.7 ± 0.23

Through the data presented in Table 5, it is possible to see that when adding the CQDs successively, both OCP and the corrosion potential are shifted to more negative potentials, confirming that the inhibitory particles act predominantly in the cathodic reaction, observed by the polarization curves (Figure 11). The decrease in the corrosion current density with the inhibitor concentration shows that the more particles there are in the corrosive medium, the more active sites on the mild steel surface are blocked by the inhibitor, thus delaying the corrosive process.^76^ These results corroborate the hypothesis that the adsorption could be physical since the positive CQDs particles interact with the cathodic sites.

These results corroborate those already reported in gravimetric and electrochemical impedance studies, showing the inhibitory potential of carbon quantum dots obtained from sugar cane bagasse.

### 3.4 Adsorption Isotherms

To analyze the adsorption process of carbon quantum dots derived from sugar cane bagasse on the mild steel surface, adsorption isotherms were studied using the Langmuir, El-Alwady, Temkin, and Flory–Huggins equations (eqs 7–11), as indicated in the equations below:
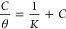
7
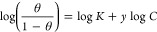
8
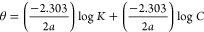
9
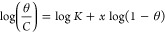
10
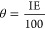
11where θ represents the degree of surface coverage, *K* is the adsorption-desorption equilibrium constant, *C* is the concentration of the inhibitor (ppm), a is the lateral interaction parameter between the adsorbed molecules, *x* is the number of adsorbed water molecules replaced by inhibitory molecules, and *y* is the number of inhibitory molecules adsorbed at each active site.

To determine which isotherm best represents the process occurring on the metal surface, linear regression was obtained using the 2 h mass loss test, and the coefficient of determination was used as a parameter (Table 6).

**Table 6:** Linear Equations Were Obtained from the Isotherms Using the 2 h Mass Loss Tests for Mild Steel in 1 mol L^–1^ HCl in the Absence and Presence of CQDs

isotherm	fit equation	*R*^2^
Langmuir	*y* = 1.07 *x* + 15.4	0.987
El-Alwady	*y* = 0.897 *x* – 1.10	0.865
Temkin	*y* = 0.452 *x* – 0.0521	0.806
Flory–Huggins	*y* = 1.065 *x* – 1.23	0.721

As shown in Table 6, the Langmuir isotherm obtained the highest coefficient of determination *R*^2^ = 0.987, while for the El-Alwady, Temkin, and Flory–Huggins isotherms, the values obtained were 0.865, 0.806, and 0.721, respectively. The data show that the Langmuir isotherm best suits the experimental data. This model assumes that adsorption occurs in specific and homogeneous locations on the surface of the adsorbent, with each site responsible for the adsorption of only a single molecule.^77^ However, because the exact molecular composition of the adsorbed particles is unknown, it is impossible to determine the value of Δ*G*_ads_.

Despite the good linear correlation (*R*^2^ = 0.987) for the Langmuir isotherm, the slope deviated a bit by one unit, suggesting a possible interaction between the adsorbed molecules or that the active site/adsorbed molecule ratio is not 1.^78−82^

From the El-Alwady model, it is possible to predict the number of inhibitory molecules occupying an active site on the surface of the metal (*y*). The value obtained for the inhibitor studied was 0.897. As the value obtained was lower than 1, it shows that each inhibitory molecule occupies more than one active site on the surface of the metal.^83^

The Flory–Huggins isotherm is associated with the number of displaced water molecules and adsorbed inhibitor molecules on the metal surface (*x*). The value of the constant *x* in eq 10 represents the number of water molecules displaced by the inhibitor. The value obtained was 1.0651, indicating that an inhibitory molecule replaced more than one water molecule.^83^ Both *x* and *y* values suggest that the CQDs are bulky.

The Temkin isotherm also showed a good correlation with the experimental data. From the Temkin equation, the value of the interaction parameter between the molecules on the surface of the electrode was calculated with *g* = −2.55. The negative value indicates repulsive lateral interactions in the adsorption layer, corroborating the hypothesis that CQDs particles are positively charged in acid solution.^83^

## 4 Adsorption Mechanism

**Figure 12:**
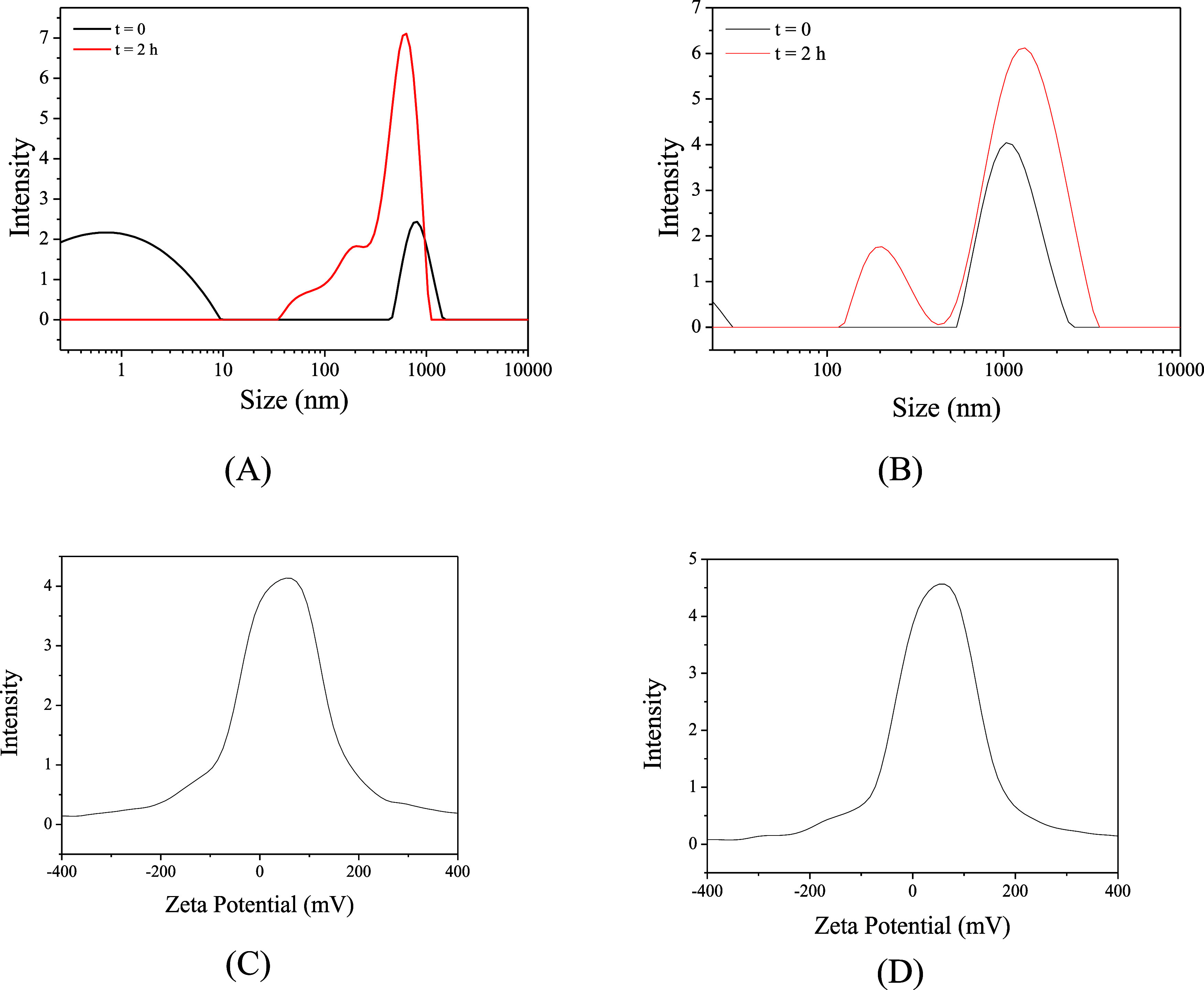
DLS analysis of produced CQDs in 1 mol L^–1^ HCl at *t*_0_ and after 2 h at concentrations of 15 ppm (A) and 100 ppm (B), and zeta potential of CQDs at concentrations of 15 ppm (C) and 100 ppm (D) at *t*_0_.

As shown, the CQDs obtained had a size distribution smaller than 10 nm in DMF. The smaller the particle size, the lower its barrier energy and the higher the particle’s surface energy resulting in high reactivity and destabilization of the colloidal system.^84,85^ This way, the nanomaterial will tend to agglomerate rapidly, reducing the system’s free energy.^84,85^ Another factor that favors the agglomeration of CQDs is their chemical composition; several functional groups and π–π stacking will also favor the interaction between CQDs, leading to cluster formation.^86^ As the reaction medium studied in this work is aqueous, the CQDs will probably agglomerate and be responsible for inhibiting corrosion. To better understand this behavior, the solution containing 1 mol L^–1^ HCl acid containing 15 and 100 ppm of CQDs was subjected to DLS and Zeta Potential analysis after 2 h and immediately after adding the CQDs to the solution (*t*_0_) (Figure 12).

As can be seen, at *t*_0_ for both concentrations (Figure 12A,B), the sample with 15 ppm presents two distribution peaks with average hydrodynamic sizes of 0.78 and 825 nm. In contrast, the 100 ppm sample presents average hydrodynamic sizes of 0.55 and 1147 nm but with different contributions. In the 15 ppm solution, a greater quantity of particles <10 nm is observed, while in the 100 ppm solution, the amount of larger particles increases significantly while smaller particles decrease. This is possibly linked to the presence of more particles favoring aggregation. After 2 h, in both samples, the presence of particles smaller than 10 nm disappears, and new groups of particles with an average size different from *t*_0_ appear. For the 15 ppm solution, the new average sizes were 55, 200, and 632 nm, while for the 100 ppm solution, the new average sizes were 200 and 1200 nm, showing that over time in both solutions, particle agglomeration occurs, being more significant for the concentration of 100 ppm due to the amount of material present. The zeta potential analysis (Figure 12C,D) for both concentrations was +15 mV, a value less than +30 mV, proving the instability of the particles and the tendency to agglomerate. This result confirms the hypothesis that CQDs particles are positively charged in an acid solution.

Relating these results to the mass loss corrosion tests, it becomes clear that the aggregation of CQDs possibly plays a crucial role in inhibiting corrosion. First, it is proven that the CQDs will immediately agglomerate in the reaction medium studied, as observed by DLS analysis (comparing with the DLS measurement in DMF) and zeta potential analysis. Second, it is proven that over time, the particles agglomerate more. The mass loss results presented in Table 1 provide strong evidence supporting the inhibition adsorption mechanism. Here, the corrosion rate decreases with increasing concentration and increases over time, indicating that the inhibition adsorption mechanism is due to the adsorption of agglomerated quantum dots on the surface of mild steel.

**Figure 13:**
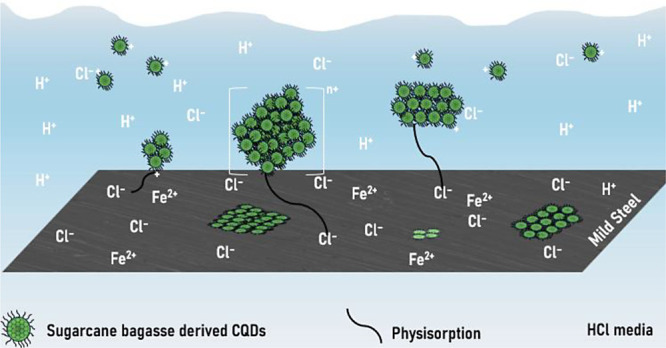
Schematic of the adsorption mechanism of N,S,Si-CQDs on Mild Steel in 1 mol L^–1^ HCl.

Figure 13 illustrates the proposed adsorption mechanism. When the MS is exposed to the corrosive HCl medium, chloride ions will adsorb on the metal surface. This will serve as a connection bridge for electrostatic interaction with the CQDs.^22^ As shown, the CQDs in the corrosive medium have a positive charge due to their functional groups, such as hydroxyl and carbonyl groups, which will allow the interaction between the MS surface and the consequent formation of a protective film.

## 5 Surface Characterization

**Figure 14:**
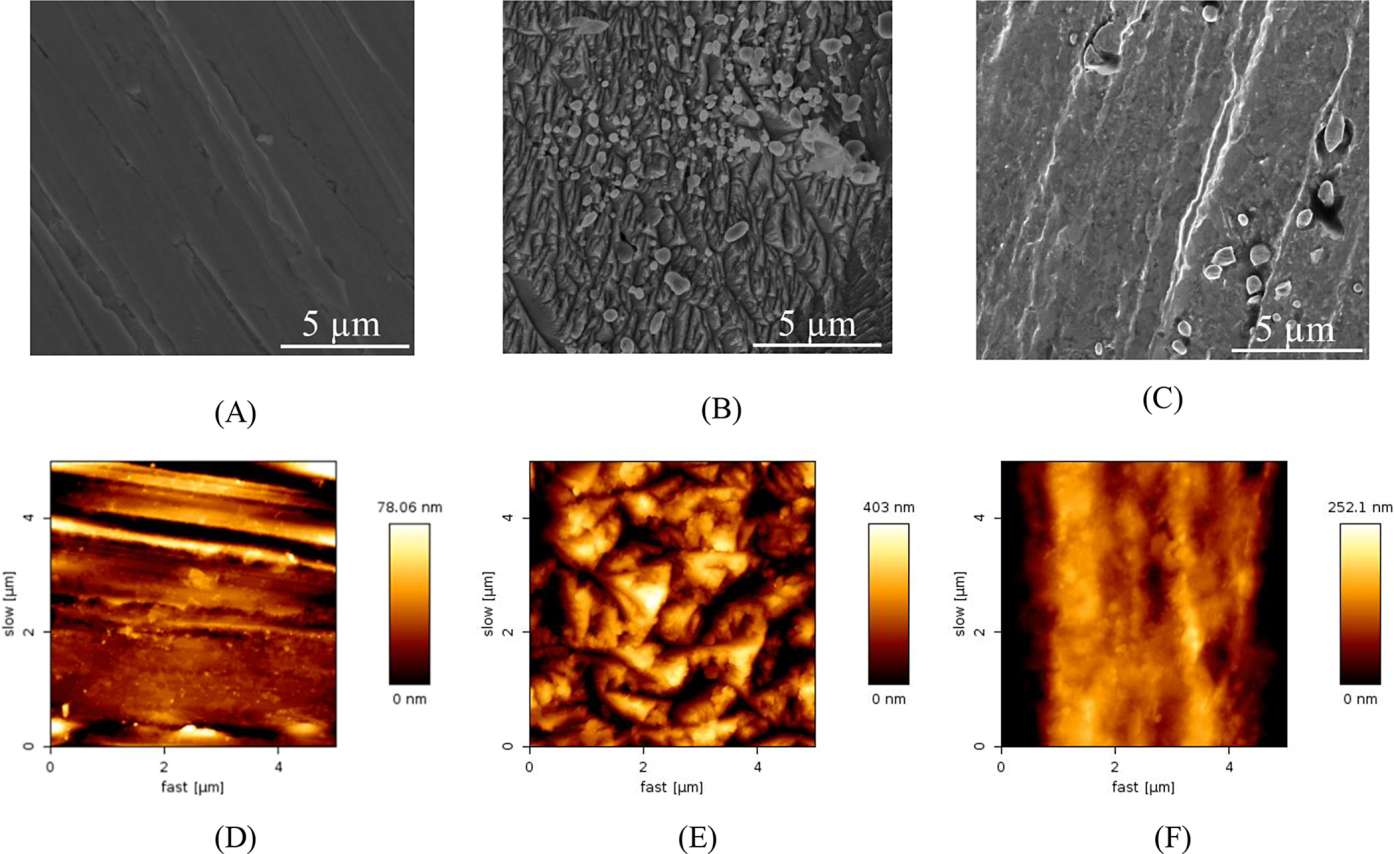
SEM and AFM measurements of Mild steel after abrading (A,D), after 2 h of immersion in 1 mol L^–1^ of HCl in the absence (B,E), and in the presence of 100 ppm of CQDs (C,F).

The surface characterization of mild steel is a crucial tool for corroboration of corrosion essays and evaluating the corrosion process. Figure 14 shows the SEM and AFM measurements, and as can be seen, after the polishing stage, mild steel presents a predominantly smooth surface with some defects in the mild steel itself (Figure 14A,D). After exposure to hydrochloric acid for two hours in the absence of the inhibitor (Figure 14B,E), the surface of the MS changes completely, presenting a rough surface showing that the iron has dissolved, corroborating the experimental results of mass loss). Finally, when exposed to the corrosive environment in the presence of 100 ppm of CQDs (Figure 14C,F) in the same period of two hours, the mild steel surface was better preserved, and it was even possible to observe a surface more similar to mild steel after abrading with a slight increase in roughness. These results corroborate the corrosion tests and confirm that the carbon quantum dots obtained are promising corrosion inhibitors.

## 6 Conclusions

The DLS, FTIR, RAMAN, and XPS results show that CQDs were successfully obtained from sugar cane bagasse, presenting a high degree of functional groups originating from the raw material itself, which is excellent as it eliminates doping and purification steps. Moreover, these groups increase the solubility of CQDs in the aqueous medium, eliminating cosolvents. These functional groups are also excellent active sites for interaction with the mild steel.

Gravimetric tests at room temperature showed that CQDs from sugar cane bagasse are efficient and remain effective over time, increasing their *IE* with time. The temperature variation study showed that the inhibitor acts through physical adsorption, while the electrochemical studies showed that the inhibitor acts preferentially on cathodic reactions. The electrochemical impedance results show an inductive loop with CQD addition, with its relaxation process occurring on the mild steel surface.

DLS and zeta potential studies prove that agglomerates of quantum dots are essential in inhibiting corrosion. The results show that CQDs agglomerate immediately when added to HCl, showing a positive charge. In contrast, a longer incubation time and high CQDs concentration result in the more significant formation of these aglomerates. In the mass loss tests due to immersion, the corrosion rate decreased with the increase in concentration and immersion period. These results show a strong correlation between inhibition efficiency and the formation of agglomerates of quantum dots.

Furthermore, when comparing the anti-corrosive efficiency of CQDs derived from sugar cane bagasse with other CQDs produced in the literature, the material obtained in this manuscript proved to be better, presenting a concentration efficiency of 96% with a concentration of only 25 ppm against 200 ppm for most works.

These results highlight a promising option for using industrial sugar cane waste, revealing the potential of biomass as a raw material to produce carbon quantum dots. The ability to transform this material into a corrosion inhibitor would offer significant advantages for the environment and industries, addressing not only corrosion-related concerns but also economic and environmental aspects.
